# The Indicative Value of Serum Tumor Markers for Metastasis and Stage of Non-Small Cell Lung Cancer

**DOI:** 10.3390/cancers14205064

**Published:** 2022-10-16

**Authors:** Chunyang Jiang, Mengyao Zhao, Shaohui Hou, Xiaoli Hu, Jinchao Huang, Hongci Wang, Changhao Ren, Xiaoying Pan, Ti Zhang, Shengnan Wu, Shun Zhang, Bingsheng Sun

**Affiliations:** 1 Department of Thoracic Surgery, National Regional Medical Center, Binhai Campus of the First Affiliated Hospital, Fujian Medical University, Fuzhou 350212, China; 2 Department of Thoracic Surgery, The First Affiliated Hospital, Fujian Medical University, 20 Chazhong Road, Fuzhou 350005, China; 3 Department of Thoracic Surgery, Tianjin Union Medical Center, Nankai University, Tianjin 300121, China; 4 Department of Occupational and Environmental Health, School of Public Health, Tongji Medical College, Huazhong University of Science and Technology, Wuhan 430030, China; 5 Department of Respiratory, The Second People’s Hospital of Linhai City, Linhai 317000, China; 6 Department of Cancer Prevention, Tianjin Medical University Cancer Institute and Hospital, Tianjin 300181, China; 7 Baodi District People’s Hospital of Tianjin, Tianjin Baodi Hospital of Tianjin Medical University, Tianjin 301000, China; 8 Medical College, Nankai University, Tianjin 300071, China; 9 Department of Thoracic Surgery, Tianjin Medical University Cancer Institute and Hospital, Tianjin 300181, China; 10 National Clinical Research Center for Cancer, Key Laboratory of Cancer Prevention and Therapy, Tianjin 300181, China

**Keywords:** non-small cell lung cancer, serum tumor markers, tumor metastasis, tumor stage, prediction

## Abstract

**Simple Summary:**

This study recruited 3272 non-small cell lung cancer (NSCLC) cases to analyze the predictive abilities of serum tumor markers (CEA, SCC-Ag, CYFRA 21-1, NSE, ProGRP, TPSA and CA199) for metastasis and clinical stage, and found that tumor marker levels may be indicative of tumor metastasis (intrapulmonary, lymphatic and distant metastasis) and stage. Increased CEA and CA199 provided an accurate prediction of intrapulmonary and distant metastasis. Increased CEA, CYFRA 21-1 and CA199 provided an accurate prediction of lymphatic metastasis and higher tumor stage. Combined detection of serum tumor markers can indicate tumor metastasis and stage in NSCLC patients.

**Abstract:**

Objective: This study aimed to explore the roles of serum tumor markers for metastasis and stage of non-small cell lung cancer (NSCLC). Methods: This study recruited 3272 NSCLC patients admitted to the Tianjin Union Medical Center and the Tianjin Medical University Cancer Institute and Hospital. The predictive abilities of some serum tumor markers (carcinoembryonic antigen (CEA), squamous cell carcinoma antigen (SCC-Ag), cytokeratin-19 fragment (CYFRA 21-1), neuron-specific enolase (NSE), pro-gastrin-releasing peptide (ProGRP), total prostate-specific antigen (TPSA) and carbohydrate antigen 199 (CA199)) for NSCLC metastasis (intrapulmonary, lymphatic and distant metastasis) and clinical stage were analyzed. Results: Tumor markers exhibited different numerical and proportional distributions in NSCLC patients. Elevated CEA, CYFRA 21-1 and CA199 levels were indicative of tumor metastasis and stage. Increased CEA and CA199 provided an accurate prediction of intrapulmonary and distant metastasis with the area under the receiver operator characteristic curve (AUC) of 0.69 both (*p* < 0.001); Increased CEA, CYFRA 21-1 and CA199 provided an accurate prediction of lymphatic metastasis with the AUC of 0.62 (*p* < 0.001). Conclusion: Combined detection of serum tumor markers can indicate tumor metastasis and stage in NSCLC patients.

## 1. Introduction

Lung cancer has been identified as one of the most common malignant tumors. In recent years, the incidence of lung cancer has gradually increased. Among different malignancies, lung cancer has the fastest-growing incidence and mortality, becoming the biggest threat to people’s health and life [1,2]. Lung cancer is divided into two types: non-small cell lung cancer (NSCLC) and small cell lung cancer (SCLC). NSCLC accounts for about 80–85% of lung cancers, including squamous cell carcinoma, adenocarcinoma, and large cell carcinoma [3]. Compared with SCLC, NSCLC cells grow and divide slowly and metastasize relatively late [4]. The early clinical symptoms of lung cancer are not obvious; thus, lung cancer is usually diagnosed at an advanced stage, and optimal treatment and surgical opportunities are lost. Thus, improving the early detection rate of lung cancer is needed to immediately adopt positive treatment measures to reduce the harm of the disease.

Tumor markers reflect the presence of the tumor, and changes in the presence and level of markers indicate the nature of the tumor. Detecting tumor markers facilitates the early diagnosis and operation of tumor development. In recent years, more serum tumor markers have been identified for the early diagnosis of lung cancer. Due to the low sensitivity and specificity of single serum tumor markers, detecting multiple tumor markers has been used to improve the sensitivity and specificity of clinical diagnosis in lung cancer patients. Therefore, the application of single tumor markers has gradually progressed to the diagnostic use of multiple markers, thus improving the positive rate of diagnosis and monitoring the development of lung cancer [5,6]. Clinically significant serum tumor markers for lung cancer include carcinoembryonic antigen (CEA), cytokeratin-19 fragment (CYFRA 21-1), neuron-specific enolase (NSE), squamous cell carcinoma antigen (SCC-Ag), pro-gastrin-releasing peptide (ProGRP), total prostate-specific antigen (TPSA) and carbohydrate antigen 199 (CA199) [7,8,9,10]. Numerous studies have reported the application of these tumor markers in the diagnosis of lung cancer. Clinical studies have also focused on the use of these markers for monitoring treatment efficacy and prognosis; furthermore, progress has been made in the application of these markers [11,12,13,14,15]. High levels of tumor markers at baseline are correlated with worse survival in stage III-IV NSCLC patients [13]. Tumor markers such as CYFRA21-1, SCC-Ag, NSE, and CEA in the serum of lung cancer patients are significantly increased, and the degree of elevation are significantly correlated with tumor invasion, clinical stage and lymph node metastasis [16].

Serum tumor markers have been used for the early diagnosis of lung cancer and the clinical practice of tumor efficacy monitoring for more than 10 years. However, confirmative studies with large clinical sample size on the consistency of various tumor markers for determining the pathology and tumor progression of lung cancer remain lacking. Thus, the purpose of this study was to explore the effectiveness of serum markers in determining tumor metastasis and stage in lung cancer patients from two clinical centers with a large sample size.

## 2. Materials and Methods

### 2.1. Patients and Control Subjects

In total, 4690 lung cancer patients admitted to Tianjin Union Medical Center from September 2016 to September 2019 and 2700 lung cancer patients admitted to Tianjin Medical University Cancer Institute and Hospital from January 2018 to September 2019 were screened as study subjects. All patients were screened using case data, relevant laboratory examination data, imaging data, and pathological examination data. Patients were determined to be all Chinese from north China and northeast China. The inclusion criteria were complete information including age, sex, smoking history, and other basic data of the patients. Patients were excluded from the study if they had other tumors, inflammation in the lungs or areas other than the lungs, or a history of chronic gastritis or ulcer in the digestive system. A total of 3272 patients with NSCLC were included in this study.

Fasting blood samples were taken for determination of lung cancer-related serum tumor markers before surgery, chemotherapy, radiotherapy, or other special treatments at the first admission. The pathological diagnosis was based on lung cancer surgery, lung puncture biopsy or tracheoscopy. Pathological diagnoses of lung cancer included squamous cell carcinoma, adenocarcinoma, adenosquamous cell carcinoma, etc. Data entry for all patients included smoking index, intrapulmonary, lymphatic and distant metastasis, and tumor stage (according to the International Association for the Study of Lung Cancer, IASLC 2015 TNM stage for lung cancer).

### 2.2. Sample Collection and Measurement

All the patients had an empty stomach the morning after the first admission without any treatment. Venous blood (5 mL) was collected from each patient to detect lung cancer-related tumor markers. The whole blood was separated into serum and cellular fractions within 2 h by centrifugation at 4000 rpm for 10 min. Serum samples were obtained after separation, and serum concentrations of tumor markers were determined. CEA, SCC-Ag and CA199 were determined by chemiluminescent microparticle immunoassay (CMIA) using an Abbott ARCHITECT I2000SR automatic chemical microparticle immune analyzer and its supporting reagents. CYFRA 21-1, NSE, ProGRP, and TPSA were determined using a Roche Elecsys 2010 automatic electrochemiluminescence immune analyzer and its supporting reagents.

### 2.3. Statistical Analysis

All tumor markers had non-normal distribution, and markers were represented by the median (P25–P75). Nonparametric tests were used to compare the concentrations of tumor markers and the smoking index between the different groups. Chi-square tests were also used to determine the distribution differences of basic information (age, sex and smoking index) and tumor markers among different groups. The Bonferroni method was used for paired comparisons. Binary logistic analysis was used to analyze the influencing factors for lung tumor metastasis, lymphatic metastasis and distant metastasis, while ordinal logistic analysis was used in examining the influencing factors for tumor stage. The two logistic analyses were divided into two steps: (1) univariate factor analysis and logistic analysis for each potential influencing factor was conducted; (2) influencing factors of *p* < 0.2 [17] in univariate analysis were included in a multivariate logistic analysis. Finally, the prediction probabilities of tumor markers with *p* < 0.05 were reassessed by logistic analysis using multivariate analysis, and the receiver operating characteristic (ROC) curves were used for joint predictions. SPSS 24.0 (IBM, Chicago, IL, USA) was used for all analyses. For two-sided tests, a *p* value less than 0.05 was considered significant.

## 3. Results

### 3.1. Demographics and Clinical Characteristics

In total, 3272 NSCLC patients were analyzed in this study. Patient characteristics are described in Table 1. The correlations of age, sex, and smoking index with tumor metastasis, and the stage of patients with NSCLC, are presented in Table 2. The percentages of intrapulmonary and lymphatic metastases were higher in male patients than in female patients. Distribution ratios of tumor stage were statistically different; the proportions of patients with stages II–IV were lower than stage I; the proportions of patients with stages II and III were lower than stage IV.

The distribution of tumor stage, intrapulmonary and lymphatic metastases in patients ≤ 61 years old were statistically different from patients > 61 years. In patients > 61 years, the number of patients with stage II and III cancers was lower than stage I. Patients with lung tumor metastasis had a higher smoking index. Smoking indexes were statistically different for patients with different tumor stages.

### 3.2. Clinical Data and Risk Factor of Tumor Metastasis and Stage in NSCLC Patients

As shown in Table 3 and Table 4, the levels of CEA, CYFRA 21-1 and NSE were significantly higher in patients with intrapulmonary, lymphatic and distant metastases when compared with those in non-metastatic patients. The levels of SCC-Ag, ProGRP and TPSA in patients with lymphatic metastasis were significantly higher than those in non-metastatic patients. The levels of CA199 in patients with lymphatic and distant metastases were significantly higher than those in non-metastatic patients. Moreover, the levels of the six tumor markers CEA, SCC-Ag, CYFRA 21-1, NSE, TPSA and CA199 were significantly different in patients with different tumor stages.

The results of the univariate analysis were summarized in Table 5. Single factors with *p* < 0.2 were included in the multivariate logistic analysis. The results of the collinearity analysis were presented in Table 6. The results of the multi-factor analysis were presented in Table 7. Binary logistic regression analysis showed that age, smoking index, CEA and CA199 were independent factors for intrapulmonary metastasis; age, CEA, CYFRA 21-1 and CA199 were independent factors for lymphatic metastasis; and age, CEA and CA199 were independent factors for distant metastasis. Ordinal logistic analysis showed that gender, age, adenocarcinoma (vs. squamous carcinoma), CEA, CYFRA 21-1, NSE and CA199 were independent factors for tumor stage.

### 3.3. The Predictions of Single and Combined Factors for Tumor Metastasis and Stage in NSCLC Patients

**Figure 1:**
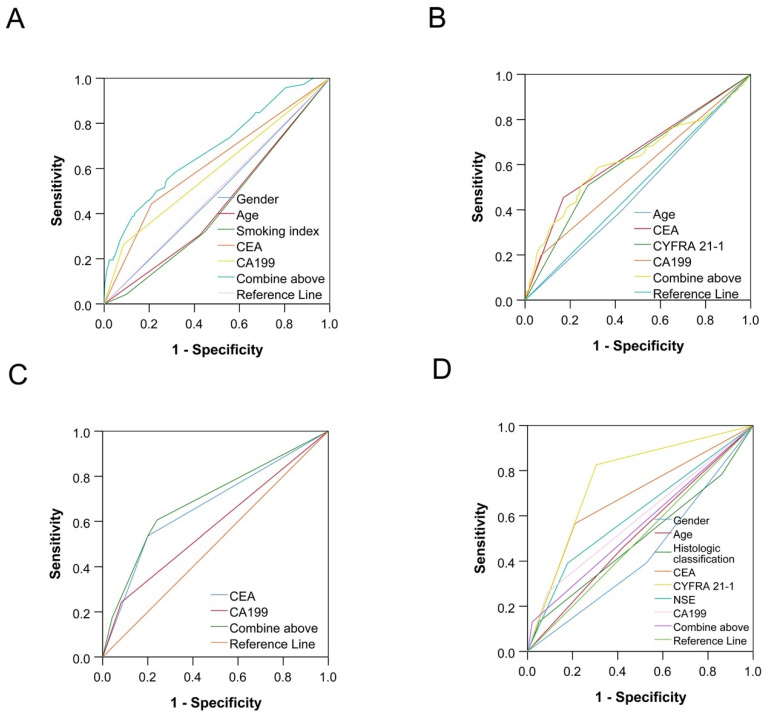
ROC curves of tumor markers for tumor metastasis and stage. (**A**) The ROC curve for gender, age, smoking index, CEA and CA199 in the diagnosis of intrapulmonary metastasis; (**B**) the ROC curve for age, CEA, CYFRA 21-1 and CA199 in the diagnosis of lymphatic metastasis. (**C**) the ROC curve for CEA and CA199 in the diagnosis of distant metastasis. (**D**) the ROC curve for gender, age, histologic classification, CEA, CYFRA 21-1, NSE and CA199 in the diagnosis of tumor stage.

Multivariate analysis of tumor markers with *p* < 0.05 was followed by a logistic analysis of prediction probability. For analysis of the influencing factors of tumor metastasis and clinical stage of NSCLC patients, the area under the ROC curve (AUC) for factors is shown in Table 8. ROC curves were used to predict lung cancer metastasis and stage, and the results were shown in Figure 1. CEA and CA199 provided an accurate prediction of intrapulmonary and distant metastasis with the AUC of 0.69 both (*p* < 0.001); CEA, CYFRA 21-1 and CA199 provided an accurate prediction of lymphatic metastasis with the AUC of 0.62 (*p* < 0.001).

## 4. Discussion

Tumor markers have been widely used in the clinical diagnosis and treatment of malignant tumors as they serve as important indicators of disease outcome monitoring. At present, varied tumor markers, such as CEA, SCC-Ag, CYFRA 21-1, NSE, are applied in diagnosing lung cancer, which can also be used to monitor metastasis and recurrence of NSCLC [18]. Although tumor markers are widely used in clinical practice, the clinical analysis and validation of these markers using a large sample size remain lacking. In this study, a large sample of lung cancer patients from two medical centers was selected to verify the accuracy of tumor markers from multiple perspectives, including predicting tumor metastasis and clinical stage. Our results support the use of these markers in clinical practice.

CEA is widely found in adult cancer tissues and has been used in the auxiliary diagnosis, efficacy observation, prognostic judgment, and recurrence prediction of cancer [19,20]. CEA elevation is common in multisystem tumors, including lung cancer [21]. Due to the non-specificity of this indicator, CEA is often used in combination with other tumor markers in clinical practice [22,23]. SCC-Ag participates in the regulation of protein decomposition during malignant transformation, and it is the preferred tumor marker for cervical squamous cell carcinoma [24,25]. Additionally, this marker is observed to increase in lung squamous cell cancers [26]. CYFRA 21-1 is highly expressed in lung squamous cell carcinoma compared with adenocarcinoma and SCLC [27]. The use of increased serum levels of CYFRA 21-1 for predicting postoperative recurrence in lung cancer patients shows good sensitivity and specificity. CYFRA 21-1 is also a highly sensitive and specific biomarker for the prediction of post-chemotherapy progression [28].

A high concentration of serum NSE is a specific marker of neuroendocrine tumors [29,30]. SCLC regulates the secretion of a variety of related enzymes, active peptides, and hormones [31]. Thus, NSE is a preferred marker for SCLC. NSE is only significantly changed in middle and advanced SCLC. NSE has been found to be related to changes in tumor growth and can be combined with clinical observations and monitoring to predict metastasis and recurrence for NSCLC [32]. ProGRP is a marker of small cell lung cancer. Serum CA199 can be used for pancreatic cancer. Auxiliary diagnostic indicators for malignant tumors such as gallbladder cancer are mainly used as indicators for disease monitoring and predicting recurrence.

In this study, we have analyzed the differences in tumor markers among patients with different metastases and tumor stages. The results showed that the levels of SCC-Ag, ProGRP and CA199 in patients with lymphatic metastasis, and the levels of CEA, CYFRA 21-1 and NSE in patients with intrapulmonary, lymphatic, and distant metastasis, were significantly higher than those patients with non-metastasis. This data indicates that the increased tumor markers significantly correlate with NSCLC metastasis [13]. Lung cancer markers have been also associated with the clinical stage of lung cancer. Tumor markers related to NSCLC, such as CEA, SCC-Ag and CYFRA 21-1, show a clear relationship with tumor stage [12,33]. The results of this study showed that there were statistical differences in the numeric levels and proportion of six tumor markers, including SCC-Ag, CEA, CYFRA 21-1, NSE, CA199 and TPSA, among patients with different tumor stages of NSCLC.

The results of risk factors showed that the patients, with increased levels of CEA, CYFRA 21-1, NSE and CA199, tended to have higher tumor stages. The risk factors for intrapulmonary metastasis were smoking index > 600, and increased levels of CEA and CA199. The risk factors for lymphatic metastasis were higher levels of CEA, CYFRA 21-1 and CA199. The risk factors for distant metastasis were elevated CEA and CA199 levels.

Combined detection of certain serum tumor markers in lung cancer patients can significantly improve diagnostic sensitivity and the roles of monitoring tumor progression [34,35]. At last, joint predictions of combined lung cancer-related tumor markers for tumor metastasis have been performed by ROC curve analyses. The result showed that the combined elevations in CEA and CA199 were also useful for the diagnoses of lymphatic metastasis and distant metastasis, respectively. These results are in accordance with previous reports [36].

## 5. Conclusions

In summary, our results suggest that the levels of CEA, SCC-Ag, CYFRA 21-1, NSE and CA199 were positively related to tumor metastasis and stage. Elevated CEA and CA199 levels in NSCLC patients are indicative of intrapulmonary and distant metastases; elevated CEA, CYFRA 21-1 and CA199 levels in patients with NSCLC are indicative of lymphatic metastasis. These tumor markers could be useful in predicting tumor metastasis in patients with NSCLC.

## Figures and Tables

**Table 1:** Basic information of the patients with NSCLC.

Patient Characteristics	Case or Median	%
Age		
median (P25–P75, year)	61 (55–67)	
Gender		
male	1812	55.4
female	1460	44.6
Smoking index		
median (P25–P75)	1.5 (0.00–600)	
non-smoking	1633	49.9
≤600 *	1009	31.2
>600	630	19.5
Intrapulmonary metastasis		
none	2656	83.8
yes	512	16.2
Lymphatic metastasis		
none	2147	67.6
yes	1031	32.4
Distant metastasis		
none	2607	81.7
yes	585	18.3
Histologic classification		
Squamous carcinoma	735	22.5
Adenocarcinoma	2354	72.0
Adenosquamous carcinoma	44	1.3
Others	139	4.2
Staging		
Ι	1997	61.3
ΙΙ	503	15.4
ΙΙΙ	175	5.4
ΙV	585	17.9

Note: * The P75 smoking index among smokers was 600.

**Table 2:** The correlation of age, sex, and smoking index with tumor metastasis, and stage of patients with NSCLC.

Variables	IM			LM			DM			Staging				
	None	Yes	*p*	None	Yes	*p*	None	Yes	*p*	Ι	ΙΙ	ΙΙΙ	ΙV	*p*
Gender (case)														
Male	1417	327	<0.001	1079	670	<0.001	1403	360	0.098	966	346	129	360	<0.001
Female	1239	185		1068	361		1204	225		1031	157 *	46 *	225 *^#&^	
Age (case)														
≤61 years	1377	221	<0.001	1111	494	0.047	1318	290	0.701	1064	206	77	290	<0.001
>61 years	1279	291		1036	537		1289	295		933	297 *	98 *	295	
Smoking index (case)														
non	1356	231	<0.001	1170	423	<0.001	1307	289	0.003	1112	178	52	289	<0.001
600	849	140		669	321		837	159		616	176	52	159	
>600	451	141		308	287		463	137		268	149 *	71 *^#^	137 *^#&^	

Note: DM, distant metastasis; IM, intrapulmonary metastasis; LM, lymphatic metastasis. Chi-square test was used to analyze. * Compared with staging Ι, adjustment *p* < 0.05; ^#^ compared with staging ΙΙ, adjustment *p* < 0.05; ^&^ compared with staging ΙΙΙ, adjustment *p* < 0.05.

**Table 3:** The difference in tumor metastasis for the tumor markers in the patients with NSCLC (CEA, SCC-Ag, CYFRA 21-1, NSE, ProGRP, TPSA and CA199).

Variables	IM			LM			DM		
	None	Yes	*p*	None	Yes	*p*	None	Yes	*p*
CEA	3.02	6.71	<0.001	2.71	5.67	<0.001	2.94	10.58	<0.001
	(1.78–5.86)	(3.18–34.55)		(1.64–4.98)	(2.91–25.93)		(1.73–5.47)	(3.43–58.54)	
SCC-Ag	0.9	0.9	0.129	0.9	1	<0.001	0.9	0.9	0.45
	(0.70–1.30)	(0.60–1.80)		(0.70–1.30)	(070–1.80)		(0.70–1.40)	(0.60–1.70)	
CYFRA 21-1	2.77	4.96	<0.001	2.59	4.6	<0.001	2.77	4.83	<0.001
	(1.92–4.56)	(2.63–10.13)		(1.85–3.95)	(2.63–10.13)		(1.92–4.47)	(2.58–12.57)	
NSE	13.32	16.5	<0.001	13.4	15.2	<0.001	13.36	16.09	<0.001
	(11.30–16.35)	(13.00–20.70)		(11.30–16.00)	(12.33–20.70)		(11.30–16.35)	(12.47–23.64)	
ProGRP	32.79	32.37	0.674	32.37	34.38	0.013	32.66	33.79	0.44
	(26.23–40.64)	(26.04–38.82)		(26.06–40.28)	(27.90–42.08)		(26.16–40.66)	(27.48–39.34)	
TPSA	45.73	50.12	0.17	44.46	56.28	<0.001	46.21	39.49	0.58
	(26.10–85.73)	(35.07–87.54)		(25.74–81.91)	(29.61–101.00)		(26.20–85.83)	(25.27–80.74)	
CA199	0.39	0.46	0.073	0.38	0.45	<0.001	0.39	0.48	<0.001
	(0.26–0.63)	(0.26–1.03)		(0.25–0.61)	(0.31–0.87)		(0.25–0.63)	(0.31–1.05)	

Note: DM, distant metastasis; IM, intrapulmonary metastasis; LM, lymphatic metastasis. Non-normal data are represented by median (P25–P75). Nonparametric test was used to analyze. Comparison of the concentrations of tumor markers between different groups was conducted by a nonparametric test.

**Table 4:** The difference in clinical stages for the tumor markers in the patients with NSCLC (CEA, SCC-Ag, CYFRA21-1, NSE, ProGRP, TPSA and CA199).

Variables	I	II	III	IV	*p*
CEA	2.59 (1.57–4.64)	4.10 (2.45–10.12) *	5.39 (3.26–15.75) *^#^	10.58 (3.43–58.54) *^#&^	<0.001
SCC-Ag	0.90 (0.70–1.20)	1.00 (0.70–2.10) *	1.10 (0.80–2.90) *^#^	0.90 (0.60–1.70) *^#&^	<0.001
CYFRA 21-1	2.50 (1.81–3.68)	4.21 (2.48–8.56) *	5.72 (3.62–14.22) *^#^	4.83 (2.58–12.57) *^#&^	<0.001
NSE	12.93 (11.07–15.60)	14.72 (12.20–18.59) *	17.45 (12.82–24.96) *^#^	16.09 (12.47–23.64) *^#^	<0.001
ProGRP	32.64 (26.15–40.76)	32.40 (26.01–39.19)	34.51 (29.14–41.78)	33.79 (27.48–39.34)	0.526
TPSA	44.37 (25.82–82.10)	56.94 (30.34–118.79) *	58.57 (45.11–128.41) *	39.49 (25.27–80.74) ^#&^	<0.001
CA199	2.31 (1.35–5.60)	2.85 (1.36–7.35) *	3.01 (1.44–8.29) *^#^	3.25 (1.43–10.72) *	<0.001

Note: Non-normal data are represented by median (P25–P75). Nonparametric test was used to analyze. Comparison of the concentrations of tumor markers between different groups was conducted by a nonparametric test. * Compared with staging I, adjustment *p* < 0.05; ^#^ compared with staging II, adjustment *p* < 0.05; ^&^ compared with staging III, adjustment *p* < 0.05.

**Table 5:** Univariate analysis of influencing factors for tumor metastasis and clinical stage in patients with NSCLC.

Variables	IM		LM		DM		Staging	
	OR (95% CI)	*p*	OR (95% CI)	*p*	OR (95% CI)	*p*	OR (95% CI)	*p*
Gender	0.64 (0.53–0.77)	<0.001	0.54 (0.47–0.63)	<0.001	0.73 (0.61–0.87)	<0.001	0.69 (0.64–0.76)	<0.001
Age	1.04 (1.03–1.05)	<0.001	1.02 (1.01–1.03)	<0.001	1.02 (1.01–1.03)	<0.001	1.01 (1.01–1.02)	<0.001
Smoking index								
non	Reference		Reference		Reference		Reference	
600	0.60 (0.48–0.75)	<0.001	0.82 (0.69–0.97)	0.019	0.60 (0.48–0.75)	<0.001	0.85 (0.78–0.94)	0.001
>600	2.00 (1.64–2.45)	<0.001	2.39 (2.00–2.86)	<0.001	1.55 (1.27–1.90)	<0.001	1.54 (1.39–1.71)	<0.001
Histologic classification								
Squamous carcinoma	Reference		Reference		Reference		Reference	
Adenocarcinoma	0.82 (0.66–1.03)	0.086	0.63 (0.74–1.62)	<0.001	0.98 (0.77–1.25)	0.873	0.63 (0.54–0.73)	<0.001
Adenosquamous carcinoma	1.33 (0.62–2.87)	0.469	1.80 (0.96–3.40)	0.068	1.55 (0.72–3.34)	0.259	1.31 (0.75–2.29)	0.338
Others	0.52 (0.29–0.93)	0.027	0.91 (0.62–1.34)	0.912	0.95 (0.55–1.64)	0.858	0.84 (0.60–1.19)	0.331
CEA	3.19 (2.66–3.82)	<0.001	3.60 (3.09–4.19)	<0.001	4.27 (3.57–5.11)	<0.001	2.40 (2.20–2.62)	<0.001
SCC-Ag	1.82 (1.49–2.22)	<0.001	2.02 (1.70–2.40)	<0.001	1.63 (1.34–2.00)	<0.001	1.52 (1.38–1.67)	<0.001
CYFRA 21-1	4.15 (3.38–5.08)	<0.001	4.28 (3.65–5.02)	<0.001	3.69 (3.04–4.47)	<0.001	2.63 (2.41–2.84)	<0.001
NSE	3.11 (2.59–3.74)	<0.001	2.59 (2.21–3.03)	<0.001	2.88 (2.41–3.44)	<0.001	1.95 (1.78–2.13)	<0.001
ProGRP	0.76 (0.18–3.17)	0.707	1.84 (1.07–3.16)	0.028	1.38 (0.55–3.51)	0.495	1.23 (0.89–1.69)	0.205
TPSA	0.92 (0.54–1.57)	0.760	1.61 (1.26–2.06)	<0.001	0.89 (0.57–1.40)	0.615	1.21 (1.05–1.39)	0.007
CA199	3.82 (2.23–6.54)	<0.001	0.32 (0.23–0.44)	<0.001	4.00 (2.53–6.31)	<0.001	2.08 (1.73–2.50)	<0.001

Note 1: DM, distant metastasis; IM, intrapulmonary metastasis; LM, lymphatic metastasis. Note 2: (1) The influencing factors of IM, LM and DM were analyzed by binary logistic analysis. Assignment of dependent variable: IM, LM and DM are all 0 = without and 1 = with. Independent variable assignment: gender (0 = male, 1 = female); age (0 = ≤61 years, 1 = >61 years); smoking index (0 = non, 1 = 1–600, 2 = ≥600); histologic classification (0 = Squamous carcinoma, 1 = Adenocarcinoma, 3 = Adenosquamous carcinoma, 4 = Others); CEA, SCC, CYFRA 21-1, NSE, ProGRP, TPSA, CA199 (0 = normal, 1 = high). (2) The influencing factors of tumor stage were analyzed by ordered logistics. Assignment of dependent variable: tumor stage (1–4 are stage Ι–ΙV, respectively); independent variable assignment: gender, age, smoking index, histologic classification and seven kinds of tumor markers are all the same as above.

**Table 6:** Collinearity examination of multifactor analysis for basic condition and tumor markers related with metastasis and stage of patients with NSCLC.

Variables	IM		LM		DM		Staging	
	T	VIF	T	VIF	T	VIF	T	VIF
Gender	0.635	1.576	0.633	1.580	0.636	1.572	0.633	1.581
Age	0.937	1.067	0.937	1.067	0.936	1.068	0.937	1.067
Smoking index	0.629	1.589	0.628	1.591	0.629	1.589	0.628	1.592
Histologic classification	0.944	1.059	0.939	1.064	-	-	0.939	1.064
CEA	0.891	1.122	0.892	1.122	0.895	1.118	0.891	1.122
SCC-Ag	0.900	1.111	0.896	1.116	0.911	1.097	0.896	1.116
CYFRA 21-1	0.863	1.159	0.737	1.356	0.872	1.147	0.737	1.357
NSE	0.975	1.025	0.976	1.025	0.980	1.020	0.976	1.025
TPSA	-	-	0.797	1.254	-	-	0.797	1.254
CA199	0.922	1.085	0.922	1.084	0.925	1.081	0.922	1.084

Note 1: DM, distant metastasis; IM, intrapulmonary metastasis; LM, lymphatic metastasis. Note 2: T, tolerance; VIF, variance inflation factor.

**Table 7:** Multivariate analysis of influencing factors for tumor metastasis and clinical stage in patients with NSCLC.

Variables	IM		LM		DM		Staging	
	OR (95% CI)	*p*	OR (95% CI)	*p*	OR (95% CI)	*p*	OR (95% CI)	*p*
Gender	0.52 (0.29–0.94)	0.031	0.85 (0.61–1.19)	0.335	0.66 (0.39–1.13)	0.117	0.68 (0.49–0.94)	0.021
Age	0.58 (0.34–0.99)	0.045	0.64 (0.48–0.83)	0.001	0.66 (0.42–1.03)	0.068	0.76 (0.59–0.98)	0.036
Smoking index								
non	Reference		Reference		Reference		Reference	
600	0.53 (0.27–1.03)	0.059	1.00 (0.70–1.42)	0.987	0.66 (0.37–1.15)	0.144	0.95 (0.67–1.34)	0.775
>600	0.28 (0.08–0.99)	0.048	1.22 (0.75–2.00)	0.413	0.45 (0.18–1.11)	0.082	1.03 (0.65–1.64)	0.889
Histologic classification								
Squamous carcinoma	Reference		Reference		Reference		Reference	
Adenocarcinoma	2.69 (0.78–9.31)	0.119	0.92 (0.62–1.38)	0.695	-	-	0.68 (0.47–0.97)	0.033
Adenosquamous carcinoma	-	-	1.58 (0.35–7.15)	0.553	-	-	1.05 (0.26–4.31)	0.948
Others	1.28 (0.20–8.12)	0.793	1.32 (0.72–2.43)	0.365	-	-	0.87 (0.49–1.54)	0.628
CEA	2.66 (1.53–4.63)	<0.001	3.08 (2.33–4.07)	<0.001	4.51 (2.86–7.12)	<0.001	2.85 (2.17–3.75)	<0.001
SCC-Ag	0.40 (0.14–1.18)	0.096	1.35 (0.95–1.93)	0.093	0.50 (0.24–1.03)	0.060	1.04 (0.74–1.45)	0.842
CYFRA 21-1	1.27 (0.73–2.22)	0.394	2.00 (1.48–2.69)	<0.001	1.06 (0.66–1.69)	0.822	2.56 (1.92–3.42)	<0.001
NSE	1.51 (0.86–2.66)	0.155	1.15 (0.84–1.57)	0.382	1.26 (0.77–2.08)	0.362	1.76 (1.33–2.34)	<0.001
TPSA	-	-	0.98 (0.72–1.32)	0.876	-	-	0.75 (0.56–1.01)	0.055
CA199	2.80 (1.49–5.17)	0.001	2.04 (1.41–2.96)	<0.001	2.20 (1.31–3.69)	0.003	2.20 (1.54–3.14)	<0.001

Notes are the same as in Table 5.

**Table 8:** Receiver operating characteristic (ROC) curves of influencing factors for tumor metastasis and clinical stage in patients with NSCLC.

Variables	IM		LM		DM		Staging	
	AUC (95% CI)	*p*	AUC (95% CI)	*p*	AUC (95% CI)	*p*	AUC (95% CI)	*p*
Gender	0.49 (0.43–0.56)	0.862	-	-	-	-	0.43 (0.35–0.52)	0.116
Age	0.44 (0.38–0.51)	0.085	0.48 (0.45–0.52)	0.362	-	-	0.52 (0.43–0.60)	0.659
Smoking index	0.43 (0.37–0.49)	0.044	-	-	-	-	-	-
Histologic classification	-	-	-	-	-	-	0.50 (0.40–0.59)	0.949
CEA	0.62 (0.55–0.69)	0.001	0.64 (0.61–0.68)	<0.001	0.67 (0.61–0.73)	<0.001	0.68 (0.59–0.76)	<0.001
CYFRA 21-1	-	-	0.62 (0.58–0.65)	<0.001	-	-	0.76 (0.70–0.83)	<0.001
NSE	-	-	-	-	-	-	0.61 (0.52–0.70)	0.013
CA199	0.59 (0.52–0.66)	0.011	0.56 (0.53–0.60)	<0.001	0.58 (0.52–0.64)	0.007	0.59 (0.49–0.68)	0.046
Combine	0.69 (0.62–0.75)	<0.001	0.62 (0.59–0.66)	<0.001	0.69 (0.63–0.75)	<0.001	0.55 (0.46–0.65)	0.205

Notes: DM, distant metastasis; IM, intrapulmonary metastasis; LM, lymphatic metastasis.

## Data Availability

Data are available upon request.

## Abbreviations

CA199, carbohydrate antigens; CEA, carcinoembryonic antigen; CMIA, chemiluminescent microparticle immunoassay; CYFRA 21-1, cytokeratin-19 fragment; DM, Distant metastasis; IM, Intrapulmonary metastasis; LM, Lymphatic metastasis; NSCLC, non-small cell lung cancer; NSE, neuron-specific enolase; ProGRP, pro-gastrin-releasing peptide; ROC, receiver operating characteristic; SCC-Ag, squamous cell carcinoma antigen; SCLC, small cell lung cancer; TPSA, total prostate-specific antigen.
